# *Weizmannia coagulans* BC99 Improves Strength Performance by Enhancing Protein Digestion and Regulating Skeletal Muscle Quality in College Students of Physical Education Major

**DOI:** 10.3390/nu16233990

**Published:** 2024-11-21

**Authors:** Li Cao, Minghan Guo, Yiqing Zhou, Jie Zhang, Shanshan Tie, Xuan Li, Pingping Tian, Ying Wu, Shaobin Gu

**Affiliations:** 1 College of Food and Bioengineering, Henan University of Science and Technology, Luoyang 471023, China; xbcaoli@163.com (L.C.); guomh321@163.com (M.G.); 15036058770@163.com (Y.Z.); zhangjie@haust.edu.cn (J.Z.); tieshanshan@haust.edu.cn (S.T.); lixuan8401@163.com (X.L.); tp2021@haust.edu.cn (P.T.); wuying2000@126.com (Y.W.); 2 Henan Engineering Research Center of Food Microbiology, Luoyang 471023, China; 3 National Demonstration Center for Experimental Food Processing and Safety Education, Luoyang 471023, China

**Keywords:** probiotic, strength performance, skeletal muscle, protein digestion, inflammation, oxidative stress

## Abstract

**Background:** The dietary proteins are one of the most important factors determining health conditions in humans. The sufficient digestion and absorption of dietary proteins in the digestive tract has positive effects on performance and recovery in sportspeople and athletes. Improving protein digestibility is a strategy for maintaining health status and optimal performance in sport and exercise activities. **Objectives:** The aim of the present study is to verify whether *Weizmannia coagulans* BC 99 (BC99) can increase muscle mass and strength. **Methods:** This randomized double-blind, controlled trial assigned 72 male college students to receive probiotics (*n* = 36, 20.25 ± 1.03 years; 179.00 ± 5.94 cm; 73.55 ± 8.73 kg, protein powder with BC99) or the placebo (*n* = 36, 20.19 ± 0.79 years; 179.25 ± 5.16 cm; 73.61 ± 8.24 kg, protein powder) for 12 weeks. At the baseline and final stages of the study, strength tests and body composition assessment were performed. Blood and stool samples were taken at the end of the 12-week intervention, and digestive enzymatic activity of stool samples, biochemical parameters, amino acids and hormone level of plasma were analyzed. **Results:** BC99 administration significantly improved strength performance, skeletal muscle mass, activity of pepsin and trypsin, the concentrations of branched chain amino acids and essential amino acids, reduced activities of creatine kinase and lactic dehydrogenase and urea nitrogen (BUN) level and increased testosterone and glucagon-like peptide-1 level in male college students. **Conclusions:** Therefore, BC99 supplementation can be an important nutritional strategy to improve strength performance, body composition, protein digestion and body metabolism in healthy young males.

## 1. Introduction

Muscle strength is associated with body composition including skeletal muscle mass and fat mass [1]. Changes in body composition are influenced by lifestyle, physical exercise and eating habits [2]. Generally, fat intake and carbohydrates were deemed as the cause of obesity, cardiovascular diseases and diabetes. The importance of protein intake seems to follow a socio-cultural trend, “carbs bad/protein good”. Indeed, an adequate intake of proteins in the diet is critical for maintaining good health and athletic performance in humans. Proteins and their hydrolysates, including amino acids, dipeptides and tripeptides, possess metabolic regulation function besides building tissues or organs [3,4]. Some studies have observed that the insufficient digestion of protein, reduction of enzyme secretion and its activity led to reduction of amino acid levels in blood [5] and affected the metabolism and function of amino acids in skeletal muscle [6]. Therefore, the digestion of food proteins in the gastrointestinal tract and amino acid metabolism are the two most important factors that maintain or improve muscle strength.

It is well established that optimal performance of athletes in some sports is associated with skeletal muscle mass and strength. Muscle protein synthesis (MPS) and breakdown (MPB) in skeletal muscle can lead to changes in skeletal muscle mass and muscle strength [7]. Reduction of skeletal muscle mass are associated with metabolic disorders [8] and aging [9].

Dietary supplements are used for improving body composition, endurance, sports performance and metabolic adaptations [10,11]. Protein and amino acids have been used for maintaining strength, recovering muscular mass and preventing nutritional deficiencies [12]. Intake of protein and amino acids can also stimulate MPS [12,13]. However, undigested protein and amino acids can be utilized by bacteria in the intestines and produce harmful metabolites including skatole and sulfide [14], and can cause gastrointestinal discomfort [15]. Thus, intervention strategies have been proposed to improve protein digestibility in the gastrointestinal tract and amino acid metabolism levels in the body.

Probiotics are living microorganisms that have positive effects on the health of the host when ingested in adequate quantities [16]. Many studies have suggested that probiotics can influence the digestion and utilization of proteins in the intestinal tract. Improvement of dietary protein digestion by probiotics can be achieved through regulation of the intestinal microbiota, increase of digestive enzyme activity and improvement of digestibility and absorption. Studies in animals have demonstrated that *Lactobacillus plantarum* GF103 and *Bacillus subtilis* B27 improved digestibility of crude protein [17]. Clinical trials have also shown that the probiotic *Bacillus coagulans* GBI-30 increases the digestion and uptake of plant proteins [18]. These results showed that probiotics can beneficially modulate digestion and utilization of proteins in the intestinal tract.

Several studies have shown that gut microbiota could influence muscle metabolism and function [19,20]. Therefore, the hypothesis of the ‘gut–muscle axis’ has been raised to study the associations between microbiota and muscle [21], and lead to some new research projects which focus on probiotics to target muscle mass and function [22,23]. Probiotic strains of commercial use mainly include the genera *Lactobacillus*, *Bifidobacterium* and *Bacillus* [24]. *Weizmannia coagulans* BC99 (BC99) is a Gram-positive, spore-forming and lactic-acid-producing probiotic strain isolated from the fecal sample of a healthy infant in Inner Mongolia [25], is used as probiotics and has exhibited its potential to improve intestinal diseases, such as irritable bowel syndrome, acute diarrhea, colitis and constipation [26]; however, the effect of BC99 on physical performance, including muscle mass and strength, has not yet been evaluated. The purpose of this study is to evaluate the effects of BC99 on physical performance and verify whether BC99 would promote protein digestion in college athletes. The hypothesis of the present study is that BC99 can increase muscle mass and strength.

## 2. Materials and Method

### 2.1. Ethics Approval and Consent to Participate

The present study was conducted at the college of food and bioengineering, Henan University of Science and Technology, China. It was conducted in accordance with the Declaration of Helsinki, and the study protocol was approved by the Research Ethics Committee of Henan University of Science and Technology (Approval code: 2004-007, date: 13 January 2024) (NCT06307821); all participants signed an approved informed consent form prior to participation.

### 2.2. Participant Characteristics

Male college students who agreed to take probiotics were recruited for this study. Those who had been exposed to antibiotics within 3 months or had a neuromuscular disorder, acute or chronic metabolic, respiratory or cardiovascular disorder, lactose intolerance or any other signs of ill health were excluded.

A total of 72 participants who met the enrollment criteria were randomly assigned to the placebo group (n = 36) and the probiotic group (n = 36). Participant characteristics and biochemical parameters are shown in Table 1. Differences between the placebo and probiotics groups were not statistically significant.

Powdered probiotics (3 g dose of milk protein concentrate with 6 × 10^9^ CFU BC99/pack) and placebo (3 g dose of milk protein concentrate/pack) were obtained from the Wecare Probiotics Co., Ltd. (Suzhou, China), and the two were indistinguishable by the naked eye. Participants were instructed to consume 8 packs (either placebo or probiotic) per day for the duration of the 12-week supplementation period. The participants were periodically monitored to ensure full compliance with the treatment of this study.

### 2.3. Experimental Design

**Figure 1:**
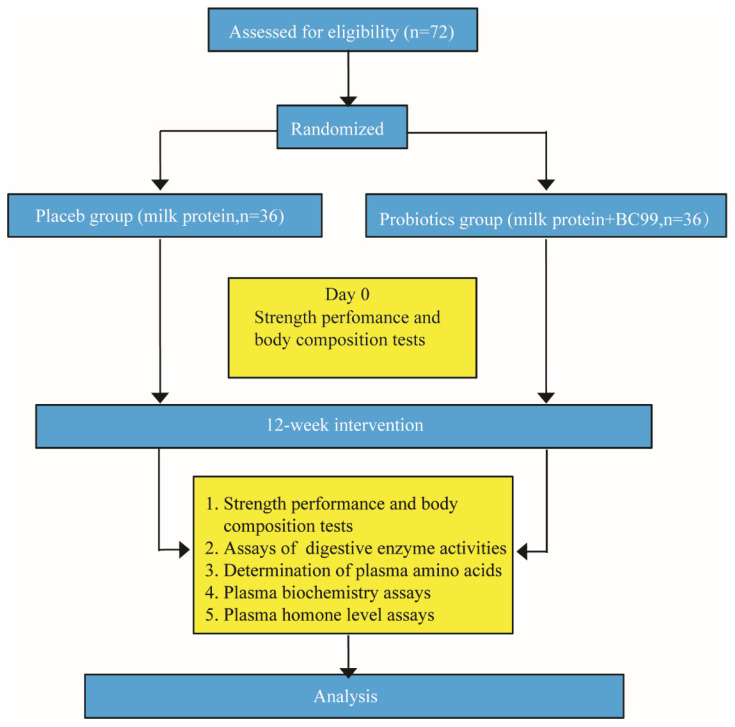
The schedule of procedure for the present study.

This study was conducted using a randomized, double-blind design. The study procedures are shown in Figure 1.

During a 12-week intervention period, all participants had an identical exercise protocol because they were from the same major.

### 2.4. Strength Performance and Body Composition

At the baseline and final stages of the study, strength tests were performed, the one repetition maximum (1RM) of the barbell bench press and squat was assessed and the number of 80%RM repetitions was scored according to previous studies with some modifications [27,28]. Body composition was assessed through InBody270 (Biospace Co., Ltd., Seoul, Republic of Korea) at the baseline and final stages of the study.

### 2.5. Enzyme Assays

At the final stages of the study, stool samples were collected using sterilized EP tubes (Nalge Nunc, Rochester, NY, USA), and stored at −80 °C until assayed. Frozen 100 mg stool samples were placed into a 2 mL centrifuge tube with 1 mL PBS, and centrifuged at 3500× *g* for 10 min at 4 °C after being homogenized. Then, the supernatant was harvested for analysis of the digestive enzyme. The activities of digestive enzymes including pepsin, trypsin and lipase were determined according to assay kit instructions (Nanjing Jiancheng Bioengineering Institute, Nanjing, China). The enzyme activities were expressed as units per milligram or gram of protein, and the amount of protein in the samples was determined using a commercially available bicinchoninic acid kit (Nanjing Jiancheng Bioengineering Institute).

### 2.6. Venous Blood Collection and Processing

At the final stages of the study, venous blood was collected after 12 h fasting, plasma samples were obtained by centrifuging at 3000 rpm for 5 min at 4 °C and then all samples were stored at −80 °C for further analysis. Blood was also taken at 3 h post-ingestion; the plasma samples were obtained for amino acid analyses according to the above method.

### 2.7. Amino Acid Determination

The Agilent 1290 Infinity ultra-high performance liquid chromatography and QTRAP^®^ 6500+ tandem triple quadrupole mass spectrometer (UHPLC-MS/MS, SCIEX Pte. Ltd., Framingham, MA, USA) were used for free amino acids analysis in the plasma sample, and quantified by isotope internal standard. A test solution was prepared according to the following steps: 50 μL of plasma sample and 50 μL of mixed solution of isotope internal standards with 16 amino acid (50 μM) were added to 400 μL of methanol/acetonitrile (*v*/*v*, 1:1) solution, separately. Then, the above solution was mixed on a vortex mixer for 1 min and left to stand for 1 h at 4 °C to allow the protein complete precipitation. Subsequently, a resulting test solution was obtained via centrifugation at 12,000 rpm for 20 min at 4 °C.

Chromatographic separation was carried out on Waters ACQUITY UPLC BEH Amide column (2.1 mm × 150 mm, 1.7 µm). Column temperature was set as 35 °C. Mobile phase A consisted of MQ water containing 0.1% (*v*/*v*) formic acid and mobile phase B was a mixture of 0.1% formic acid-acetonitrile. The flow rate and injection volume were 300 µL/min and 2 µL, respectively. The gradient profile is as follows: Initial, 85% B; 1 min, 85% B; 3 min, 50% B; 11.5 min, 40% B; 12 min, 75% B.

The MS conditions were as follows: The electro-spray ionization source (ESI) was operated using the positive mode with capillary voltage of 4.5 kV and an ion source temperature of 500 °C. The flow rates of ion source gas1, ion source gas2 and curtain gas were set at 55 L/h, 50 L/h and 30 L/h, respectively.

### 2.8. Biochemistry Analysis

The biochemical parameters in plasma including lactate dehydrogenase (LDH) and creatine kinase (CK) were measured using the commercial kit according to the manufacturer’s protocol (Nanjing Jiancheng Bioengineering Institute).

Myoglobin (Mb) and blood urea nitrogen (BUN) were measured using automatic biochemical analyzer (Shanghai Kehua Experimental Equipment Co., Ltd., Shanghai, China) at the Health Management Center, the Hospital of Henan University of Science and Technology.

### 2.9. Hormone Assays

The levels of testosterone and glucagon-like peptide-1 (GLP-1) in plasma were determined using a commercially available kit (Nanjing Jiancheng Bioengineering Institute).

### 2.10. Statistical Analysis

Student’s *t*-test and the Mann–Whitney test were used to compare quantitative variables between the two groups at baseline. All clinical and laboratory parameters were compared between the treatment groups (placebo and probiotic) and over time (baseline, and 12 week) using generalized linear models. *p*-values < 0.05 were considered statistically significant. All calculations were performed using IBM SPSS Statistics version 22.0.

## 3. Results

### 3.1. Effect of W. coagulans BC99 on Strength Performance

As shown in Table 2, strength performance was compared prior to and following the intervention. There were no significant differences in the bench press and squat between the two groups at the baseline. After a 12-week intervention, the number of 80%RM repetitions in bench press and squat in the probiotic group was significantly higher than that of the placebo group, while no significant differences were observed between the two groups after the 1RM test of bench press and squat. Moreover, compared with that in the baseline stage of the study, the 1RM test of bench press and squat in both the placebo and probiotic group in the final stage of study was higher (*p* < 0.05).

### 3.2. Effect of W. coagulans BC99 on Body Composition

Body composition changes were also compared prior to and following the intervention. After a 12-week intervention, compared with the placebo group, the probiotic group showed a significant increase in muscle mass and fat free mass (*p* < 0.05), a significant decrease in fat mass (*p* < 0.05) and a decreasing trend in body weight; however, the difference was not statistically significant (*p* = 0.07). Compared with that in the baseline stage, body composition in the final stage was not significantly different in the placebo group. However, compared with that in the baseline stage, muscle mass, fat-free mass showed a significant increase (*p* < 0.05), and reductions in fat mass (*p* < 0.01) in the probiotic group. The results are presented in Table 3.

### 3.3. Effect of W. coagulans BC99 on Enzymatic Activity of Stool Samples

**Figure 2:**
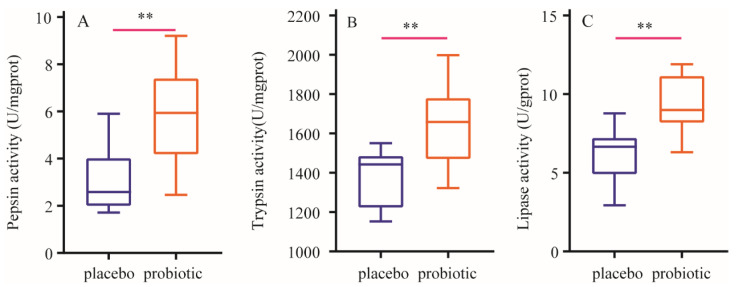
Changes in enzymatic activity of stool samples. (**A**): Changes in pepsin activity. (**B**): Changes in trypsin activity. (**C**): Changes in lipase activity. Data are expressed as mean ± SD. ** means the difference is significant at the 0.01 level.

After a 12-week intervention, compared with those in the placebo group, the activities of pepsin, trypsin and lipase significantly increased (*p* < 0.01) in the probiotic group. The results are presented in Figure 2.

### 3.4. Effect of W. coagulans BC99 on Plasma Concentration of Amino Acids

To investigate whether BC99 intervention increased the plasma concentration of amino acids by enhancing digestibility, 22 amino acids were analyzed (Table 4). The concentrations of alanine, arginine, Gamma-aminobutyric acid (GABA), glutamine, isoleucine, leucine, taurine, and tyrosine were significantly higher in the probiotic group than those in the placebo group. Compared with those in the placebo group, the concentrations of proline and valine tended to increase in the probiotic group, although the increase was not significant. In particular, the concentrations of branched chain amino acids (BCAA), including isoleucine and leucine, and essential amino acid (EAA) were significantly higher in the probiotic group than those in the placebo group. Compared to the placebo group, the concentrations of BCAA and EAA were 1.61 and 1.27 times higher in the probiotic group, respectively.

### 3.5. Effect of W. coagulans BC99 on Plasma Biochemical Parameters

After a 12-week intervention, compared with those of the placebo group, a statistically significant decrease in the activities of LDH (*p* < 0.05), CK (*p* < 0.01) and the concentration of BUN (*p* < 0.05) of the probiotic group was observed. There is no significant difference in the concentration of Mb between the two groups. The results are presented in Table 5.

### 3.6. Effect of W. coagulans BC99 on Plasma Hormone Level

**Figure 3:**
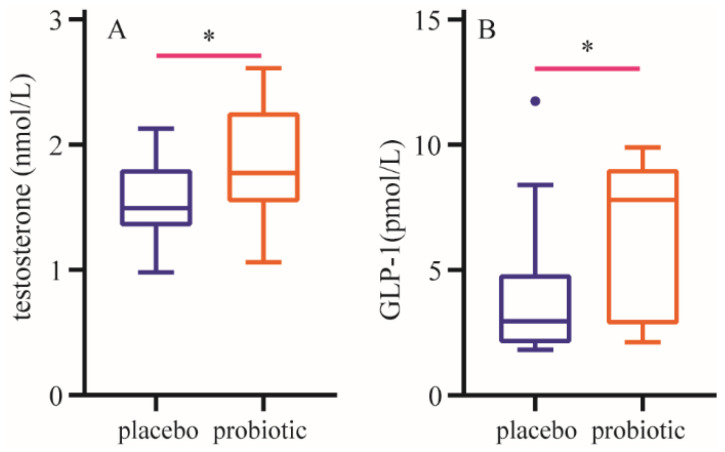
Changes in plasma hormone level. (**A**): Changes in plasma testosterone level. (**B**): Changes in t glucagon-like peptide-1 level. Data are expressed as mean ± SD. * means the difference is significant at the 0.01 level.

After a 12-week intervention, compared with those in the placebo group, plasma testosterone and GLP-1 levels were significantly increased in the probiotic group (Figure 3).

## 4. Discussion

In this study, we evaluate whether BC99 may obtain optimal athletic performance by improving protein digestion and amino acid metabolism in male students of sport education major. As we expected, BC99 significantly increased strength performance in male college students (Table 2). Notably, we observed that protein supplementation (placebo group) also significantly improved strength performance from pre-test to post-test. It is possible that protein supplementation stimulates muscle anabolism [29,30], then improved skeletal muscle mass and performance.

Several studies have shown that protein supplementation contributes to strength recovery and improvement of run performance and muscle mass [31,32]. In contrast, some studies found that protein supplementation had no efficacy for strength and performance [33,34,35]. These discrepancies can be associated with the digestibility of proteins in the gastrointestinal tract besides protein types, protein dose and the metabolic status of host. Overall, this study provides evidence that BC99 exhibited good efficacy in improvement of strength performance in the male students.

Skeletal muscle accounts for about 40% of body weight in humans. Skeletal muscle mass affects muscle strength [36] and superior athletic performance [37]. Skeletal muscle is a highly plastic tissue, being able to respond to numerous environmental cues, such as exercise and nutrition [38]. Such a response to environmental and physiological changing conditions can lead to changes in skeletal muscle mass, function and composition. In health, muscle mass is maintained by the precisely regulated muscles MPS and MPB [7]. In this study, the significant differences between the placebo and probiotic groups in muscle mass, fat mass and fat-free mass were observed (Table 3), suggesting that increase in muscle quality may have contributed to improvement of strength performance after BC99 intervention.

It has been suggested that free essential amino acid (EAA) compositions stimulated muscle protein synthesis more than the same profile and amount of EAAs in dietary protein [39] and that the higher protein digestion and amino acid absorption were more effective for stimulating MPS [39,40,41], indicating that enhancing the digestion rate of dietary protein can facilitate the release of free amino acids into the blood circulation system, increase the bioavailability of dietary protein and stimulate MPS.

We subsequently evaluated the activity of proteases including pepsin and trypsin in stool samples and the concentration of amino acid in plasma. The results showed that BC99 increased the activity of pepsin and trypsin and concentration of amino acid; specifically, levels of BCAA and EAA were significantly increased. The increase in plasma amino acid concentration seems to be, at least partly, attributed to the higher protein digestion after ingestion of BC99 compared with the single protein supplement. Clinical studies have shown that BCAA can promote MPS [42], and EAA can also effectively stimulate MPS [43]. Muscle protein synthesis is the metabolic basis for increased muscle mass and physical function [44]. Together with the above results, we concluded that the probiotic BC99 could enhance muscle protein anabolism by facilitating digestion and absorption of dietary protein. In addition, we also found that the ingestion of BC99 increased activity of lipase; these results provided evidence that BC99 could also improve digestion of other nutrients besides protein in the diet, and BC99 supplementation may be an important strategy to improve digestive function for patients with indigestion.

BUN is a fatigue-related biomarker. BC99 reduced BUN level. LDH is an enzyme which catalyzes conversion of pyruvate to lactic acid [45], and creatine kinase can convert creatine into the high-energy phosphocreatine molecule [46]. The excessive production of LDH and creatine kinase leads to muscle damage [47]; thus, both are biomarkers of muscle damage. In this study, we observed that BC99 intervention attenuated CK and LDH levels, and decreased BUN. These results indicated that BC99 could improve endurance, reduce fatigue and decrease muscle damage. Despite Mb being important in maintaining oxygen consumption and tension generation in skeletal muscle, the Mb content between two groups had no significant difference in this study.

A continuous turnover of protein (synthesis and breakdown) maintains the functional integrity and quality of skeletal muscle. Hormones are important regulators of this remodeling process. Anabolic hormones stimulate human muscle growth mainly by increasing protein synthesis or by decreasing protein breakdown. Both testosterone and glucagon-like peptide-one (GLP-1) are critical hormones relating to skeletal muscle protein metabolism. Subnormal testosterone levels led to unfavorable changes in body composition such as reducing muscle mass and strength, as well as increasing fat mass [48]. Testosterone supplementation increased muscle mass by inhibiting protein degradation in older men [49]. Some evidence suggests that testosterone supplementation attenuates the decrease in muscle mass and grip strength [49,50], increased lean body mass and leg and arm muscle strength [51]. GLP-1 is a peptide hormone with 30 amino acids and affects muscle mass and its function [52,53]. GLP-1 can augment MPS in skeletal muscle of older humans [54].

Previous studies have suggested gut microbiota affect the hormone level of the host [55]. Probiotics possess a health-promoting effect by increasing intestinal bacterial diversity and producing numerous metabolites [56]. A recent study showed that supplementary probiotic changed hormone levels and metabolic parameters of the host [57]. In our study, administration of BC99 for 12 weeks significantly increased testosterone and GLP-1 levels compared to that in the placebo group. It is possible that BC99 increases MPS by regulating hormone changes.

The study contributes to building the scientific foundation for using the probiotic BC99 as a potential tool for improving muscle protein synthesis and enhancing exercise capacity.

## 5. Conclusions

The present study demonstrated that BC99 intervention can significantly increase bench press and squat 1RM and 80%RM times, increase muscle mass and fat-free mass, decrease fat mass and increase the activities of pepsin, trypsin and lipase and the concentrations of alanine, arginine, GABA, glutamine, isoleucine, leucine, taurine and tyrosine, particularly, the concentrations of BCAA and EAA. In addition, BC99 intervention significantly decreased the activities of LDH and CK and the concentration of BUN, and increased testosterone and GLP-1 levels in plasma. These findings suggest that BC99 had positive effects on strength performance by regulating body composition, protein digestion and absorption of amino acids, metabolism of skeletal muscle-related biochemical parameters and hormone levels. Therefore, the probiotic BC99 has significant market value in various groups of individuals, from athletes to patients suffering from obesity and even sarcopenia. However, further studies are required to understand dose response and the underlying mechanisms and best practices to advance the application of BC99 as functional products.

## Figures and Tables

**Table 1:** Participant characteristics and biochemical parameters.

	Placebo (n = 36)	Probiotic (n = 36)
Age (years)	20.25 ± 1.03	20.19 ± 0.79
Height (cm)	179.00 ± 5.94	179.25 ± 5.16
Weigh (kg)	73.55 ± 8.73	73.61 ± 8.24
AST (mmol/L)	16.63 ± 9.23	15.14 ± 4.77
ALT (mmol/L)	35.53 ± 9.21	32.06 ± 8.65
TG (mmol/L)	0.97 ± 0.66	0.99 ± 0.39
TC (mmol/L)	3.49 ± 0.44	3.76 ± 0.48
Glucose(mmol/L)	4.93 ± 0.31	4.91 ± 0.29
Feces occult blood	ND	ND

Values are presented as mean ± SD. There were not statistically significant differences between the placebo and probiotics groups. AST—aspartate aminotransferase; ALT—alanine aminotransferase; TG—triglycerides; TC—total cholesterol; ND—Not Detected.

**Table 2:** Changes in strength performance in the placebo and probiotic groups at baseline and at the end of the 12-week intervention.

		Placebo		Probiotics	
		Baseline	End	Baseline	End
Bench press	1RM (kg)	68.06 ± 12.32	76.14 ± 9.04 *	70.14 ± 9.74	78.27 ± 10.29 *
	80%RM (times)	11.42 ± 3.54	10.07 ± 2.36	11.31 ± 3.09	11.41 ± 1.84 ^▲^
Squat	1RM (kg)	114.86 ± 21.51	131.90 ± 23.20 *	121.57 ± 17.65	133.65 ± 18.84 *
	80%RM (times)	10.33 ± 5.39	10.91 ± 2.96	10.54 ± 4.14	13.04 ± 4.01 ^▲^

Values are presented as mean ± SD. * means the difference between baseline and study end is significant at the 0.05 level. ^▲^ means the difference between placebo and probiotic groups is significant at the 0.05 level.

**Table 3:** Body composition variables in the placebo and probiotic groups at baseline and at the end of the 12-week intervention.

	Placebo		Probiotics	
	Baseline	End	Baseline	End
Muscle mass	34.44 ± 3.78	34.15 ± 2.93	34.47 ± 3.34	36.16 ± 3.12 *^▲^
Fat mass	11.01 ± 3.41	9.90 ± 3.55	10.91 ± 3.07	8.31 ± 2.18 **^▲^
Fat-free mass	61.28 ± 4.78	59.89 ± 4.05	61.25 ± 5.69	64.77 ± 4.59 *^▲^
Body weight	73.55 ± 8.73	69.94 ± 6.95	73.61 ± 8.24	70.08 ± 7.18
BMI	22.26 ± 2.14	21.67 ± 2.12	22.55 ± 2.13	21.95 ± 1.94

Values are presented as mean ± SD. * means the difference between baseline and study end is significant at the 0.05 level. ** means the difference between baseline and study end is significant at the 0.01 level. ^▲^ means the difference between placebo and probiotic groups is significant at the 0.05 level. BMI—Body Mass Index.

**Table 4:** Plasma concentrations of 22 amino acids (μmol/L).

Amino Acids	Placebo (n = 36)	Probiotic (n = 36)	*p* Value
Alanine	100.45 ± 5.02	119.32 ± 8.39	0.001
Arginine	74.30 ± 5.75	107.43 ± 12.13	0.000
Asparagine	36.16 ± 3.34	40.53 ± 2.84	0.210
Aspartic acid	7.18 ± 1.15	7.76 ± 0.39	0.273
Cysteine	7.19 ± 1.16	7.78 ± 0.40	0.261
GABA	0.41 ± 0.09	0.59 ± 0.08	0.004
Glutamate	1589.43 ± 251.86	1704.56 ± 431.67	0.008
Glutamine	486.67 ± 32.71	542.75 ± 25.63	0.585
Glycine	107.29 ± 10.67	105.34 ± 11.19	0.764
Histidine	87.89 ± 8.82	87.60 ± 7.49	0.951
Isoleucine	75.89 ± 7.26	90.68 ± 14.11	0.001
Leucine	156.03 ± 22.01	192.79 ± 19.41	0.014
Lysine	229.17 ± 48.41	240.14 ± 25.52	0.108
Methionine	29.45 ± 2.74	32.64 ± 3.89	0.132
Phenylalanine	55.28 ± 2.05	53.47 ± 5.23	0.449
Proline	118.92 ± 9.57	132.49 ± 12.34	0.059
Serine	83.33 ± 11.38	83.15 ± 15.97	0.983
Taurine	22.85 ± 1.51	25.51 ± 2.19	0.035
Threonine	190.11 ± 13.40	205.05 ± 28.42	0.271
Tryptophan	38.75 ± 5.11	37.54 ± 4.69	0.678
Tyrosine	38.54 ± 1.67	33.07 ± 2.51	0.001
Valine	120.32 ± 9.28	131.25 ± 8.96	0.065
BCAA	350.49 ± 39.83	564.18 ± 29.06	0.000
EAA	963.65 ± 65.47	1222.31 ± 65.83	0.000
Total AA	3643.83 ± 254.01	4092.35 ± 495.51	0.077

Data are expressed as mean ± SD.

**Table 5:** Changes in plasma biochemical parameters in the placebo and probiotic groups.

	Placebo (n = 36)	Probiotic (n = 36)
LDH (U/L)	2036.90 ± 273	1327.18 ± 368 *
CK (U/mL)	0.19 ± 0.04	0.09 ± 0.03 **
BUN (mmol/L)	5.44 ± 1.05	4.96 ± 0.89 *
Mb (ng/mL)	1.74 ± 0.33	1.60 ± 0.28

Data are presented as mean ± SD. * means the difference between baseline and study end is significant at the 0.05 level. ** means the difference between baseline and study end is significant at the 0.01 level.

## Data Availability

The original contributions presented in the study are included in the article, further inquiries can be directed to the corresponding authors.

## References

[1] Nonaka K., Murata S., Shiraiwa K., Abiko T., Nakano H., Iwase H., Naito K., Horie J. (2018). Effect of skeletal muscle and fat mass on muscle strength in the elderly. Healthcare.

[2] Moscatelli F., De Maria A., Marinaccio L.A., Monda V., Messina A., Monacis D., Toto G., Limone P., Monda M., Messina G. (2023). Assessment of lifestyle, eating habits and the effect of nutritional education among undergraduate students in southern italy. Nutrients.

[3] Fuchs C.J., Hermans W.J.H., Holwerda A.M., Smeets J.S.J., Senden J.M., Kranenburg J., Gijsen A.P., Wodzig W.K.H.W., Schierbeek H., Verdijk L.B. (2019). Branched-chain amino acid and branched-chain ketoacid ingestion increases muscle protein synthesis rates in vivo in older adults: A double-blind, randomized trial. Am. J. Clin. Nutr..

[4] Bröer S., Bröer A. (2017). Amino acid homeostasis and signalling in mammalian cells and organisms. Biochem. J..

[5] Lee S., Jo K., Jeong H.G., Yong H.I., Choi Y.S., Kim D.J., Jung S. (2021). Freezing-then-aging treatment improved the protein digestibility of beef in an in vitro infant digestion model. Food Chem..

[6] Paulusma C.C., Lamers W.H., Broer S., Lamers W.H., Broer S., Graaf S.F.J. (2022). Amino acid metabolism, transport and signalling in the liver revisited. Biochem. Pharmacol..

[7] Jang L.G., Choi G., Kim S.W., Kim B.Y., Lee S., Park H. (2019). The combination of sport and sport-specific diet is associated with characteristics of gut microbiota: An observational study. J. Int. Soc. Sports Nutr..

[8] Kelley D.E., Goodpaster B., Wing R.R., Simoneau J.A. (1999). Skeletal muscle fatty acid metabolism in association with insulin resistance, obesity, and weight loss. Am. J. Physiol..

[9] Ebert S.M., Dyle M.C., Bullard S.A., Dierdorff J.M., Murry D.J., Fox D.K., Bongers K.S., Lira V.A., Meyerholz D.K., Talley J.J. (2015). Identification and Small Molecule Inhibition of an Activating Transcription Factor 4 (ATF4)-dependent Pathway to Age-related Skeletal Muscle Weakness and Atrophy. J. Biol. Chem..

[10] Mohr A.E., Jäger R., Carpenter K.C., Kerksick C.M., Purpura M., Townsend J.R., West N.P., Black K., Gleeson M., Pyne D.B. (2020). The athletic gut microbiota. J. Int. Soc. Sports Nutr..

[11] Maughan R.J. (1999). Nutritional ergogenic aids and exercise performance. Nutr. Res. Rev..

[12] Jäger R., Kerksick C.M., Campbell B.I., Cribb P.J., Wells S.D., Skwiat T.M., Purpura M., Ziegenfuss T.N., Ferrando A.A., Arent S.M. (2017). International society of sports nutrition position stand: Protein and exercise. J. Int. Soc. Sports Nutr..

[13] Holwerda A.M., Paulussen K.J.M., Overkamp M., Goessens J.P.B., Kramer I.F., Wodzig W.K.W.H., Verdijk L.B., Loon L.J.C. (2019). Dose-dependent increases in whole-body net protein balance and dietary protein-derived amino acid incorporation into myofibrillar protein during recovery from resistance exercise in older men. J. Nutr..

[14] Blachier F. (2023). Amino acid-derived bacterial metabolites in the colorectal luminal fluid: Effects on microbial communication, metabolism, physiology, and growth. Microorganisms.

[15] Wu G.Y. (2016). Dietary protein intake and human health. Food Funct..

[16] Sanders M.E., Merenstein D.J., Reid G., Gibson G.R., Rastall R.A. (2019). Probiotics and prebiotics in intestinal health and disease: From biology to the clinic. Nat. Rev. Gastroenterol. Hepatol..

[17] Zhang R., Zhou M., Tu Y., Zhang N.F., Deng K.D., Ma T., Diao Q.Y. (2016). Effect of oral administration of probiotics on growth performance, apparent nutrient digestibility and stress-related indicators in Holstein calves. J. Anim. Physiol. Anim. Nutr..

[18] Keller D., Van Dinter R., Cash H., Farmer S., Venema K. (2017). *Bacillus coagulans* GBI-30, 6086 increases plant protein digestion in a dynamic, computer-controlled in vitro model of the small intestine (TIM-1). Benef. Microbes.

[19] Grosicki G.J., Fielding R.A., Lustgarten M.S. (2018). Gut microbiota contribute to age-related changes in skeletal muscle size, composition, and function: Biological basis for a gut-muscle axis. Calcif. Tissue Int..

[20] Lustgarten M.S. (2019). The role of the gut microbiome on skeletal muscle mass and physical function: 2019 update. Front. Physiol..

[21] Bindels L.B., Delzenne N.M. (2013). Muscle wasting: The gut microbiota as a new therapeutic target?. Int. J. Biochem. Cell Biol..

[22] Rezaee N., Rahmani-Nia F., Delfan M., Ghahremani R. (2021). Exercise training and probiotic supplementation effects on skeletal muscle apoptosis prevention in type-I diabetic rats. Life. Sci..

[23] Prokopidis K., Giannos P., Kirwan R., Prokopidis K., Giannos P., Kirwan R., Ispoglou T., Galli F., Witard O.C., Triantafyllidis K.K. (2023). Impact of probiotics on muscle mass, muscle strength and lean mass: A systematic review and meta-analysis of randomized controlled trials. J. Cachexia Sarcopenia Muscle.

[24] Soares M.B., Martinez R.C.R., Pereira E.P.R., Balthazar C.F., Cruz A.G., Ranadheera C.S., Sant’Ana A.S. (2019). The resistance of and strains with claimed probiotic properties in different food matrices exposed to simulated gastrointestinal tract conditions. Food Res. Int..

[25] Zhu M., Zhu J., Fang S., Zhao B. (2024). Complete genome sequence of Heyndrickxia (Bacillus) coagulans BC99 isolated from a fecal sample of a healthy infant. Microbiol. Resour. Ann..

[26] Chaudhari K., Mohan M., Saudagar P., Sable C., Shinde S., Bedade D. (2022). In vitro and in vivo evaluation of probiotic potential and safety assessment of Bacillus coagulans SKB LAB-19 (MCC 0554) in humans and animal healthcare. Regul. Toxicol. Pharmacol..

[27] Caldwell L.K., DuPont W.H., Beeler M.K., Post E.M., Barnhart E.C., Hardesty V.H., Anders J.P., Borden E.C., Volek J.S., Kraemer W.J. (2018). The Effects of a korean ginseng, GINST15, on perceptual effort, psychomotor performance, and physical performance in men and women. J. Sports Sci. Med..

[28] Polotow T.G., Souza-Junior T.P., Sampaio R.C., Okuyama A.R., Ganini D., Vardaris C.V., Alves R.C., McAnulty S.R., Barros M.P. (2017). Effect of 1 repetition maximum, 80% repetition maximum, and 50% repetition maximum strength exercise in trained individuals on variations in plasma redox biomarkers. J. Strength Cond. Res..

[29] Cruz-Jentoft A.J., Dawson Hughes B., Scott D., Sanders K.M., Rizzoli R. (2020). Nutritional strategies for maintaining muscle mass and strength from middle age to later life: A narrative review. Maturitas.

[30] Landi F., Calvani R., Tosato M., Martone A.M., Ortolani E., Savera G., D’Angelo E., Sisto A., Marzetti E. (2016). Protein intake and muscle health in old age: From biological plausibility to clinical evidence. Nutrients.

[31] Cintineo H.P., Arent M.A., Antonio J., Arent S.M. (2018). Effects of protein supplementation on performance andrecovery in resistance and endurance training. Front. Nutr..

[32] Huang W.C., Chang Y.C., Chen Y.M., Hsu Y.J., Huang C.C., Kan N.W., Chen S.S. (2017). Whey protein improves marathon-induced injury and exercise performance in elite track runners. Int. J. Med. Sci..

[33] Forbes S.C., Bell G.J. (2019). Whey protein isolate supplementation while endurance training does not alter cycling performance or immune responses at rest or after exercise. Front. Nutr..

[34] Williamson E., Kato H., Volterman K.A., Suzuki K., Moore D.R. (2019). The effect of dietary protein on protein metabolism and performance in endurance-trained males. Med. Sci. Sports Exerc..

[35] D’Lugos A.C., Luden N.D., Faller J.M., Akers J.D., McKenzie A.I., Saunders M.J. (2016). Supplemental protein during heavy cycling training and recovery impacts skeletal muscle and heart rate responses but not performance. Nutrients.

[36] Janssen I., Baumgartner R.N., Ross R., Rosenberg I.H., Roubenoff R. (2004). Skeletal muscle cutpoints associated with elevated physical disability risk in older men and women. Am. J. Epidemiol..

[37] Tsekoura M., Kastrinis A., Katsoulaki M., Billis E., Gliatis J. (2017). Sarcopenia and its impact on quality of life. Adv. Exp. Med. Biol..

[38] Stewart C., Rittweger J. (2006). Adaptive processes in skeletal muscle: Molecular regulators and genetic influences. J. Musculoskelet. Neuronal Interact..

[39] Paddon-Jones D., Sheffield-Moore M., Katsanos C.S., Zhang X.J., Wolfe R.R. (2005). Differential stimulation of muscle protein synthesis in elderly humans following isocaloric ingestion of amino acids or whey protein. Exp. Gerontol..

[40] Devries M.C., Phillips S.M. (2015). Supplemental protein in support of muscle mass and health: Advantage whey. J. Food Sci..

[41] Dangin M., Boirie Y., Garcia-Rodenas C., Gachon P., Fauquant J., Callier P., Ballevre O., Beaufrere B. (2001). The digestion rate of protein is an independent regulating factor of postprandial protein retention. Am. J. Physiol. Endocrinol. Metab..

[42] Sharples A.P., Hughes D.C., Deane C.S., Saini A., Selman C., Stewart C.E. (2015). Longevity and skeletal muscle mass: The role of IGF signalling, the sirtuins, dietary restriction and protein intake. Aging Cell.

[43] Church D.D., Hirsch K.R., Park S., Kim I.-Y., Gwin J.S., Pasiakos S.M., Wolfe R.R., Ferrando A.A. (2020). Essential amino acids and protein synthesis: Insights to maximizing the muscle and whole-body response to feeding. Nutrients.

[44] Wolfe R.R. (2006). The underappreciated role of muscle in health and disease. Am. J. Clin. Nutr..

[45] Cho H.D., Lee J.H., Jeong J.H., Kim J.Y., Yee S.T., Park S.K., Lee M.K., Seo K.I. (2016). Production of novel vinegar having antioxidant and anti-fatigue activities from *Salicornia herbacea* L. J. Sci. Food Agr..

[46] Xu C., Lv J.L., Lo Y.M., Cui S.W., Hu X., Fan M. (2013). Effects of oat β-glucan on endurance exercise and its anti-fatigue properties in trained rats. Carbohyd. Polym..

[47] Oh H.A., Kim D.E., Choi H.J., Kim N.J., Kim D.H. (2015). Anti-fatigue effects of 20(S)-protopanaxadiol and 20(S)-protopanaxatriol in mice. Biol. Pharm. Bull..

[48] Kruse R., Petersson S.J., Christensen L.L., Kristensen J.M., Sabaratnam R., Ørtenblad N., Andersen M., Højlund K. (2020). Effect of long-term testosterone therapy on molecular regulators of skeletal muscle mass and fibre-type distribution in aging men with subnormal testosterone. Metabolism.

[49] Ferrando A.A., Sheffield-Moore M., Yeckel C.W., Gilkison C., Jiang J., Achacosa A., Lieberman S.A., Tipton K., Wolfe R.R., Urban R.J. (2002). Testosterone administration to older men improves muscle function: Molecular and physiological mechanisms. Am. J. Physiol. Endocrinol. Metab..

[50] Sinha-Hikim I., Cornford M., Gaytan H., Lee M.L., Bhasin S. (2006). Effects of testosterone supplementation on skeletal muscle fiber hypertrophy and satellite cells in community-dwelling older men. J. Clin. Endocrinol. Metab..

[51] Caminiti G., Volterrani M., Iellamo F., Marazzi G., Massaro R., Miceli M., Mammi C., Piepoli M., Fini M., Rosano G.M. (2009). Effect of long-acting testosterone treatment on functional exercise capacity, skeletal muscle performance, insulin resistance, and baroreflex sensitivity in elderly patients with chronic heart failure a double-blind, placebo-controlled, randomized study. J. Am. Coll. Cardiol..

[52] Katayama T., Takada S., Masaki Y., Kinugawa S., Matsumoto J., Furihata T., Fukushima A., Yokota T., Okita K., Tsutsui H. (2016). The activation of glucagon-like peptide-1 improves the mitochondrial abnormalities in skeletal muscle and exercise intolerance in heart failure mice. J. Card. Fail..

[53] Hong Y., Lee J.H., Jeong K.W., Choi C.S., Jun H.S. (2019). Amelioration of muscle wasting by glucagon-like peptide-1 receptor agonist in muscle atrophy. J. Cachexia Sarcopenia Muscle.

[54] Tuddenham S., Sears C.L. (2015). The intestinal microbiome and health. Curr. Opin. Infect. Dis..

[55] He S., Li H., Yu Z.H., Zhang F., Liang S., Liu H., Chen H., Lü M.H. (2021). The Gut microbiome and sex hormone-related diseases. Front. Microbiol..

[56] Falcinelli S., Rodiles A., Hatef A., Picchietti S., Cossignani L., Merrifield D.L., Unniappan S., Carnevali O. (2018). Influence of probiotics administration on gut microbiota core. J. Clin. Gastroenterol..

[57] Okuka N., Milinkovic N., Velickovic K., Polovina S., Sumarac-Dumanovic M., Minic R., Korčok D., Djordjevic B., Ivanovic N.D. (2024). Beneficial effects of a new probiotic formulation on adipocytokines, appetite-regulating hormones, and metabolic parameters in obese women. Food Funct..

